# Effects of oral intake fruit or fruit extract on skin aging in healthy adults: a systematic review and Meta-analysis of randomized controlled trials

**DOI:** 10.3389/fnut.2023.1232229

**Published:** 2023-08-04

**Authors:** Haoying Li, Lu Wang, Jinhong Feng, Lijuan Jiang, Jingping Wu

**Affiliations:** ^1^Clinical Medical College, Chengdu University of Traditional Chinese Medicine, Chengdu, Sichuan, China; ^2^Department of Medical Cosmetology, Hospital of Chengdu University of Traditional Chinese Medicine, Chengdu, China

**Keywords:** skin aging, fruit, fruit extract, oral, randomized controlled trials, meta-analysis

## Abstract

**Background:**

In recent years, oral various fruits or supplements of fruits natural extracts have been reported to have significant anti-aging effects on the skin (1, 2), However, despite many studies on this topic, there is often no clear evidence to support their efficacy and safety. In this paper, we present a comprehensive review and Meta-analysis of the evidence for the safety and efficacy of oral fruits and fruits extracts in improving skin aging.

**Methods:**

Four databases, Pubmed, Embase, Web of Science, and Cochrane Library (CENTRAL), were searched for relevant literature from 2000–01 to 2023–03. Seven randomized controlled trials (RCTs) of fruit intake or fruit extracts associated with anti-skin aging were screened for Meta-analysis.

**Results:**

Compared to placebo, oral intake of fruit or fruit extracts showed significant statistical differences in skin hydration and transepidermal water loss (TEWL), with a significant improvement in skin hydration and a significant decrease in TEWL. No significant statistical difference was observed in minimal erythema dose (MED), overall skin elasticity (R2), or wrinkle depth, and no evidence of significant improvement in skin condition was observed.

**Conclusion:**

Meta-analysis results suggest that consume administration of fruits or fruit extracts significantly enhances skin hydration and reduces transcutaneous water loss, but there is insufficient evidence to support other outcome recommendations, including minimal erythema dose (MED), overall skin elasticity(R2), and wrinkle depth.

**Systematic Review Registration:**

PROSPERO (york.ac.uk), identifier CRD42023410382.

## Introduction

Keeping skin soft and beautiful is a common desire, especially for women. Dry skin can accentuate wrinkles and make a person look older. As the largest organ of the body, the skin is the barrier between the cells and the external environment, covering most of the body (3). Regarding the mechanism of skin aging, it has been shown to be related to damage to collagen and elastic fibers by matrix metalloproteinases, extracellular matrix degradation, abnormal fibroblast function, oxidative stress and cell proliferation, apoptosis and inflammatory response, and light-induced DNA damage (4–6). Pigmentation, dryness, loss of elasticity and deepening of wrinkles are all significant changes in skin aging. Topical cosmetics are the most basic form of skin care (7, 8). Their advantages include high safety of topical application, but they act only on the skin surface, have limited effects, and uncomfortable skin reactions caused by topical agents, such as sensory reactions or subjective sensations (no signs of inflammatory events), may still be encountered after application (9–11). For example, facial skin is usually more sensitive than other parts of the body and can feel tingling and burning in those who use dermatological agents. At the same time, the skin, as living tissue, requires nutrients to remain healthy (12). The required nutrients come mainly from food and not only from topical preparations. Over the past 20 years, the trend toward achieving “beauty from within” has grown, leading individuals to adopt a healthier lifestyle by consuming nutrient-rich foods (13). Consequently, there has been a growing interest in methods that use dietary supplements or functional foods to delay or improve skin aging. Fruits, as an important component of a daily diet, contain a rich array of antioxidants, anti-inflammatory substances, vitamins, minerals, and polyphenolic compounds. They play a positive role in skin antioxidation, anti-inflammation, wrinkle reduction, and skin brightening. Consequently, there has been significant interest in the potential of consuming fruits or fruit extracts to slow down skin aging (14, 15). The aim is to restore the deepest layers of the skin, effectively improving surface appearance. This leads to the question: can fruits or fruit extracts improve skin aging, and if so, how specifically do they contribute? It has been reported that a large number of fruits or fruit extracts have beneficial effects on skin moisturization, anti-wrinkle properties, and removal of pigmentation, helping to make the skin appear younger (12, 13, 16). However, despite the importance of individual fruits or fruit extracts for skin care, there is a lack of research on their anti-aging effects. Many such randomized controlled trials (RCTs) often yield confusing and inconsistent results, presenting a challenge for consumers. Scientific evidence for the anti-aging effects of fruits or fruit extracts is generally scarce, usually stemming from *in vitro* or uncontrolled *in vivo* studies. Furthermore, few studies have provided sufficient and convincing scientific validation of their efficacy and safety.

Therefore, the purpose of this study is to conduct a detailed and Systematic review of the research on the intake of fruits and fruit extracts and their anti-skin aging effects, through extensive literature searches. By conducting Meta-analysis on randomized controlled trials, we aim to understand their efficacy and safety. This will lead to a better scientific understanding of the relationship between the intake of fruits or fruit extracts and skin aging, and its application in daily skincare.

## Methods

This article follows the PRISMA (Reporting Guidelines for Systematic Evaluation and Meta-analysis) guidelines regarding accreditation, selection, eligibility, and inclusion. The study protocol was registered in advance in the PROSPERO database (No. CRD42023410382). (17).

### Search strategy

Four databases, Pubmed, Embase, Web of Science, and Cochrane Library (Central Database), were searched for relevant literature from 2000–01 to 2023–03. Because according to the “results by year” function of Pubmed, the skin health of orally consumed fruits or fruit extracts has been extensively studied only in the last 23 years. A computerized search of the relevant literature was performed for the following terms: (1) skin aging or skin condition or skin hydration; (2) fruit or fruit extracts. These search terms were adapted accordingly for each database. Supplementary Table S1 provides a comprehensive description of the search method. In this paper, only English RCTs were selected.

### Eligibility criteria

Studies were selected when they met all of the following inclusion criteria:

1. Study type. Only parallel group randomized controlled trials (RCTs) assessing skin hydration, minimal erythema dose (MED), skin elasticity, wrinkle depth, or transepidermal water loss (TEWL) in human subjects were included. Such trials can control for participant differences by randomization and parallel group design, reduce bias, and introduce a control group taking placebo or low-dose supplements to eliminate placebo effects. The published paper must be a peer-reviewed full-text article.
2. Type of participant. Healthy individuals of any gender or race between the ages of 18 and 70 years were included. The main criteria for exclusion were as follows: (A) pregnancy, breastfeeding, and various metabolic, cardiovascular, or liver diseases; (B) serious skin-related diseases such as atopic dermatitis, psoriasis, and vitiligo; and (C) regular consumption of any food, drug, or other supplement that affects skin conditions, including health foods, antioxidant supplements, retinoids, steroids, and any other hormonal products.
3. Type of intervention. Randomized controlled trials comparing fruit or fruit extracts with placebo or lower doses of the same fruit or fruit extract were eligible. Studies were eligible when the same protocol was used in the test and control groups, regardless of any additional lifestyle interventions used. Studies using different protocols between the test and placebo groups were excluded.
4. The seven included studies involved multiple ethnic groups, with a large proportion of Asians, so in the Meta-analysis study, ethnic subgroup analyses were conducted, grouping Asians (including Koreans, Japanese, and Chinese) and non-Asians (including Americans and Italians). Both Asian and non-Asian subgroups were included in the outcome indicators of interest in this study.

### Study selection

Three reviewers (H.L., L.W.and J.W.) independently screened titles and abstracts. Full texts were imported into Endnote (Version X9, Clarivate Analytics) for further review when they were deemed potentially eligible by the initial screening process. The full texts were then independently evaluated against the aforementioned inclusion criteria by reviewers (H.L. and L.W. or J.W.) to determine their eligibility for inclusion in the study. Throughout the process, whenever any question arose, we consulted with other researchers (J.F. and L.J.) or referenced relevant literature and books for clarification.

### Data extraction

The characteristics of each included study are documented in Supplementary Table S2, including details of the study such as author(s), country and year of publication, characteristics of the study population (age, sex and health status), study duration, intervention content of each group, daily dose, the form of applications, testing environment [°C room temperature (R.T.) and % relative humidity (R.H.)], the start and end time of the study, the measurement instruments used, and the skin sites assessed. The safety of the intervention was assessed by comparing adverse events (AEs), treatment-related adverse effects (TEAs), and treatment-related discontinuations (TAW) in the intervention and placebo groups.

The other collected data, such as the baseline and endpoint measure (average) and its variability SD (standard deviation) or SE (standard error), were input into an Excel spreadsheet. Five outcomes were evaluated: skin hydration, minimum erythema dose (MED), overall skin elasticity (R2), transcutaneous water loss (TEWL), and wrinkle depth. When multiple measurement sites were provided in the study, facial and arm test areas commonly used by females were preferred as the measurement sites for assessing moisturizing effects. When data from different racial populations were provided, subgroup analysis would be performed, and studies from different races would be considered as independent studies. The study data with the longest observation period, particularly those with multiple time points of measurement results, were selected for analysis.

During the data collection process, whenever any issues arose, we consulted with other researchers to discuss and resolve them.

### Quality assessment

Three reviewers (H.L. and L.W.or J.F.) evaluated the risk of bias (ROB) in the randomized controlled trials (RCTs) using the Cochrane Collaboration’s assessment tool (18), which includes seven criteria: selection biases (SB), performance biases (PB), detection biases (DB), attrition biases (AB), reporting biases (RB), and other biases in the trial design or methodology. Examples of other biases include changes in life habits during the study, unstable conditions in test rooms that may affect the measurement results, and potential conflicts of interest. Each study was assessed for each of these criteria and rated as “low risk,” “high risk,” or “unclear.” ROB was considered low when the study adequately reported methods without potential biases according to the Cochrane Collaboration’s guidelines (18). Conversely, high ROB was assigned when the method used in the study could not remove underlying biases. If there was insufficient information to make a determination, or if the information provided was inadequate, the trial was labeled with an“unclear risk of bias.”

### Statistical analysis

#### Evaluation of overall effect size

The Meta-analysis of seven randomized controlled trials (RCTs) included in the analysis was performed using Review Manager 5.4 (19). The standardized mean differences (SMDs) of continuous variables were calculated using a random effects model in statistical analysis. The mean difference was obtained by subtracting the baseline value from the endpoint value. If changes in standard deviation (SDs) from baseline were not reported, the following formula based on the baseline and endpoint SDs of the treatment and placebo groups can be used to calculate the SD value:

SD=(SD_baseline^2+SD_final^2)−2×Corr×SD_baseline×SD_final.

As baseline-endpoint correlation coefficients (Corr) were not reported in these studies, a Corr value of 0.5 was used (20), which is a value calculated in most articles with sufficient data and ranges from 0.4 to 0.6.

In addition, if an RCT does not compare with a placebo group and only compares the effects of low-dose and high-dose interventions, the high-dose group is considered as the intervention group, and the low-dose group is considered as the placebo group. The effect size of skin hydration, minimal erythema dose (MED), skin elasticity, transepidermal water loss (TEWL), and wrinkle depth were expressed in standard mean difference (SMD) with a 95% confidence interval (CI), as not all included studies reported these five outcomes using the same device and unit of measurement. *p* < 0.05, indicating statistical significance (21).

#### Evaluation of heterogeneity

Higgins I (*I*^2^) was used to estimate heterogeneous statistics in the legend of each forest plot. The *I*^2^ index represents the proportion of observed differences in effect sizes attributable to heterogeneity rather than sampling errors in the total variation between studies (22). For instance, an *I*^2^ of 0% indicates that the entire difference in effect sizes is due to sampling error, while an *I*^2^ of 100% implies that the variation in effect sizes is due entirely to true effects. *I*^2^ values of 0–25%, 25–50%, 50–75%, and 75–100% are generally considered low, moderate, high, and very high heterogeneity, respectively (23).

## Results

### Identification of researches

**Figure 1:**
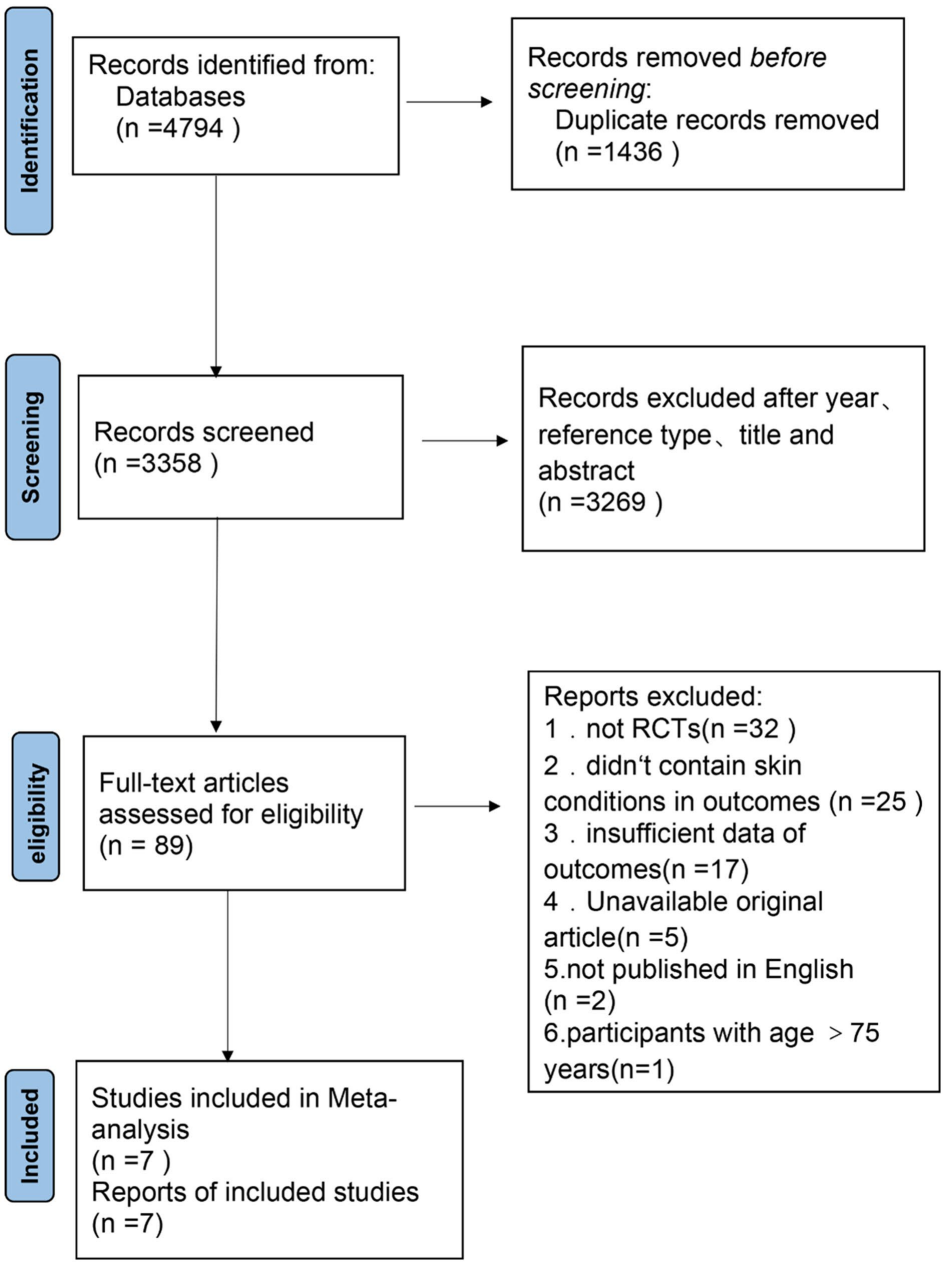
Flow diagram for study selection process.

A total of 4,794 references (Figure 1) were retrieved, including 795 from PubMed, 962 from Embase, 2,251 from Web of Science, and 786 from Cochrane Library. After screening, 7 randomized controlled trials were included in the Systematic review and Meta-analysis.

### Risk of bias within researches

Considering that Nobile 2022 involved participants from both China and Italy, studies conducted with participation from different ethnic groups are considered as separate studies.

**Figure 2:**
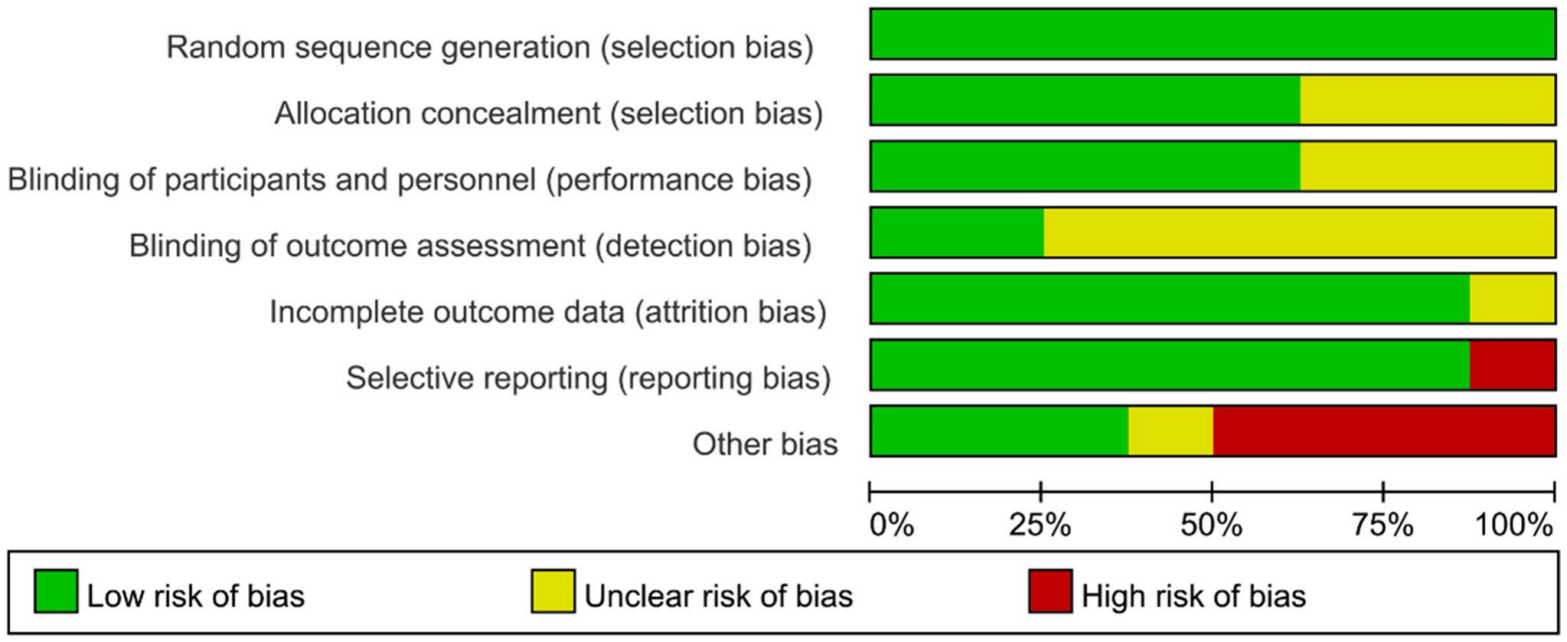
Summary of review authors’ judgments for each risk of bias domain.

The review of every ROB item presented as percentages across the entire selected research as per the judgment of the researcher was displayed in Figure 2, and the judgment for every trial was described in Supplementary Figure S1. All studies have fully reported randomization, including the use of computer-generated randomization schedules and random number tables. Therefore, the selection bias of all studies is considered to be at low risk. Five studies (63%) reported a clear process of blinding for both participants and outcome assessors, while 36% of the studies had an unclear risk of bias. Two studies (25%) were identified as having low risk of detection bias, while the remaining studies had an unclear risk of bias. Attrition bias was low risk in 7 research works (88%), and 1 research (12%) was unclear. The risk of reporting bias was high in one study (12%), while concrete data were provided and trials were pre-registered in a publicly available trial registry in seven studies (88%). Four studies (50%) were identified as having a high risk of “other biases,” with commonly observed factors including potential conflicts of interest, maintenance of participants’ living habits, and insufficient details regarding physical environmental conditions.

### Research features

Supplementary Table S2 summarizes the main characteristics of each study. Seven studies were included in a Meta-analysis to evaluate the effect of oral intake of fruits or fruit extracts on improving skin aging. Among the seven trials included in the Meta-analysis, two involved Asian participants (Japanese and Korean). One study consisted of participants from China and Italy, and three trials were conducted among U.S. participants. The primary participants were women between the ages of 30 and 70 with visible signs of skin aging, such as wrinkles and dryness, at baseline levels.

### Skin hydration

Skin hydration levels were assessed in five studies (*n* = 341), including a study conducted in both China and Italy, which were considered as two independent studies. Three studies focused on Asian populations (24–26), and three studies included non-Asian populations (27, 28). Four studies examined the effects of different fruit extract formulations, including capsules and tablets (24–27), while one study involved direct ingestion of avocado (28). These studies lasted 8–12 weeks and evaluated skin hydration levels.

**Figure 3:**
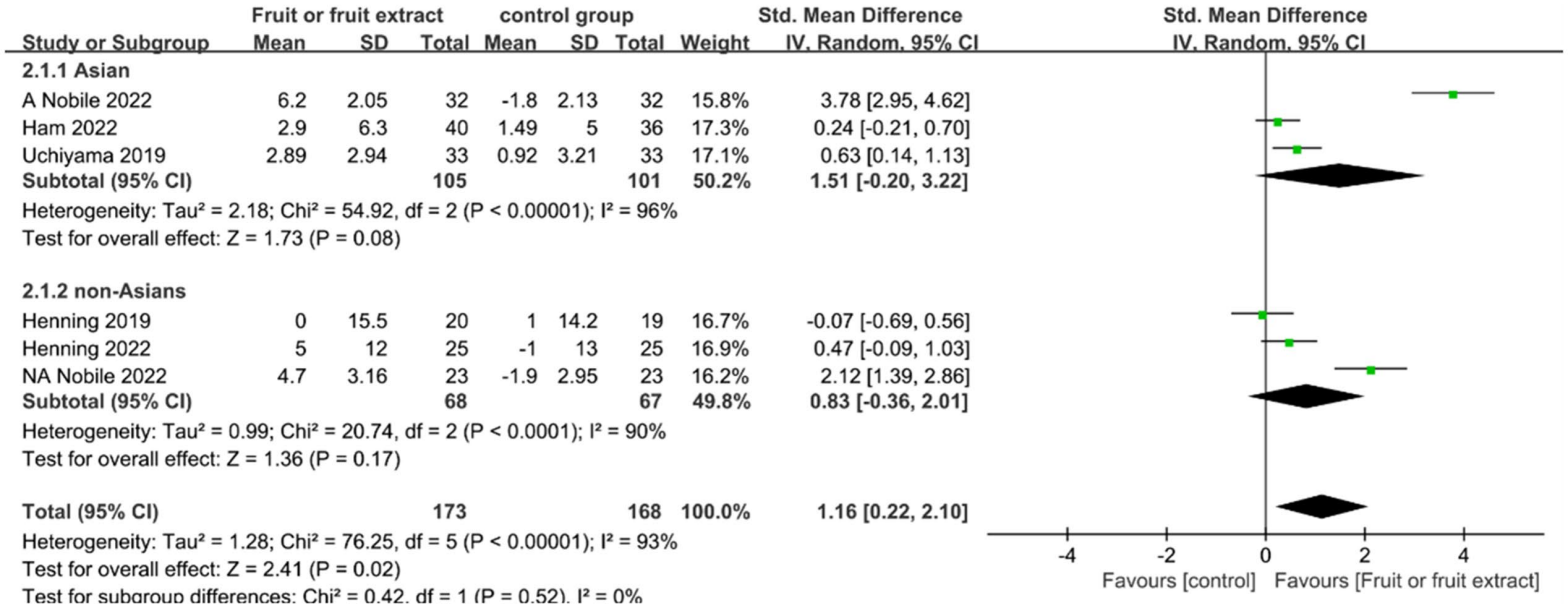
Forest plot of comparison: Fruit or fruit extract vs. placebo on skin hydration (SMD). The details of each study are reported in Supplementary Table S2. CI, confidence interval.

Figure 3 displays the forest plot of a Meta-analysis of six studies, which estimates the difference in skin hydration between intervention and placebo groups.

The results of the Meta-analysis of six studies showed no significant improvement in skin hydration in the Asian group after ingestion of fruit or fruit extract (SMD = 1.51; 95% CI: -0.2-3.22, *p* = 0.08); and no significant difference in the non-Asian group compared to the placebo group after ingestion of fruit or fruit extract (SMD = 0.83; 95% CI: −0.36- 2.01, *p* = 0.41). However, the ability of ingesting fruits or fruit extracts to promote skin hydration was supported by statistical evidence based on the overall effect size summary analysis (SMD = 1.16; 95% CI: 0.22–2.10, *p* = 0.02).Overall, both the Asian group (*I*^2^ = 96%) and the non-Asian group () exhibited a high degree of heterogeneity. Sensitivity analysis using the one-by-one exclusion method found that the heterogeneity significantly decreased(*I*^2^ = 23%，*I*^2^ = 36%) when the study by Nobile 2022 was excluded, with no change in the pooled effect result for skin hydration (SMD = 0.35; 95%CI: 0.07–0.62,*p* = 0.01) (Supplemental Figure S2); the heterogeneity mainly originated from Nobile 2022, which may be due to the skin measurements differed in location, the study measured skin moisture on the back, which is less exposed to the external environment, while other studies measured skin hydration on the face and forearm, which are more exposed.

### Skin elasticity

Skin elasticity was monitored in three studies (*n* = 225) lasting from 8 to 12 weeks. One study was carried out in both China and Italy and was considered as two independent studies. Two studies were included in the Asian population group (24, 25), whereas the other two studies were included in the non-Asian population group (24, 27). The four studies focused on different fruit extract formulations (including capsules and tablets).

**Figure 4:**
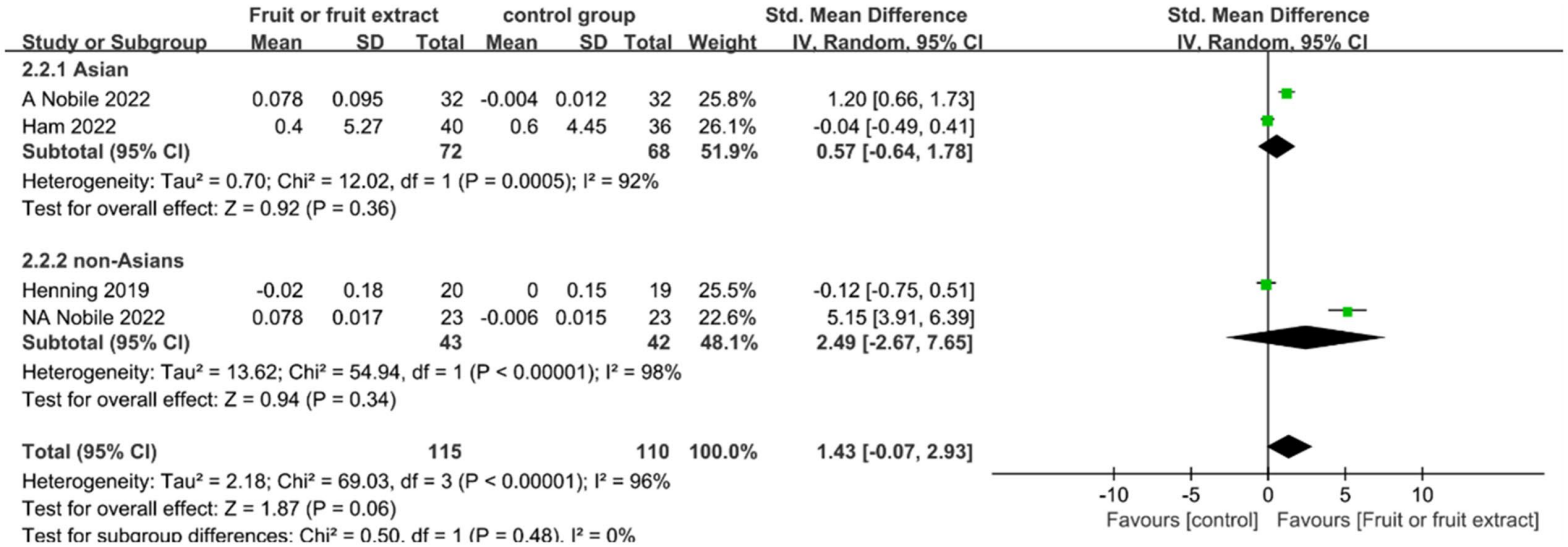
Forest plot of comparison: Fruit or fruit extract vs. placebo on skin elasticity (SMD). The details of each study are reported in Supplementary Table S2. CI, confidence interval.

Figure 4 displays the forest plot of a Meta-analysis of four studies, which estimates the difference in skin elasticity between intervention and placebo groups.

The Meta-analysis of four studies (*n* = 225) revealed no significant effect of consuming fruits and fruit extracts on increasing skin elasticity compared to placebo (SMD = 1.43; 95% CI: −0.07-2.93, *p* = 0.06) (Figure 4). No significant correlation was observed in both the Asian and non-Asian groups.In the Meta-analysis of these two subgroups, the overall study exhibited high heterogeneity (I^2^ of 92 and 98%, respectively), which may be attributed to different sources of fruit extracts, including red-orange complex extract, *Citrus sinensis* extracts (PTE), and pomegranate extracts, different extraction methods such as standardized extracts and 50% ethanol extracts, as well as the differences in body parts of the tested subjects (back, eye contour, and arm).

### Wrinkle depth

Four studies (*n* = 280) evaluated wrinkle depth over a period of 8 to 12 weeks. One of the trials included both Chinese and Italian participants, and studies with different ethnic groups were considered as independent studies. Therefore, three studies were included in the Asian group (24–26), while two studies were included in the non-Asian group (24, 29). Among them, the focus of four studies (24–26) was on different formulations of fruit extracts, including capsules and tablets, while one study (29) directly consumed mangoes.

**Figure 5:**
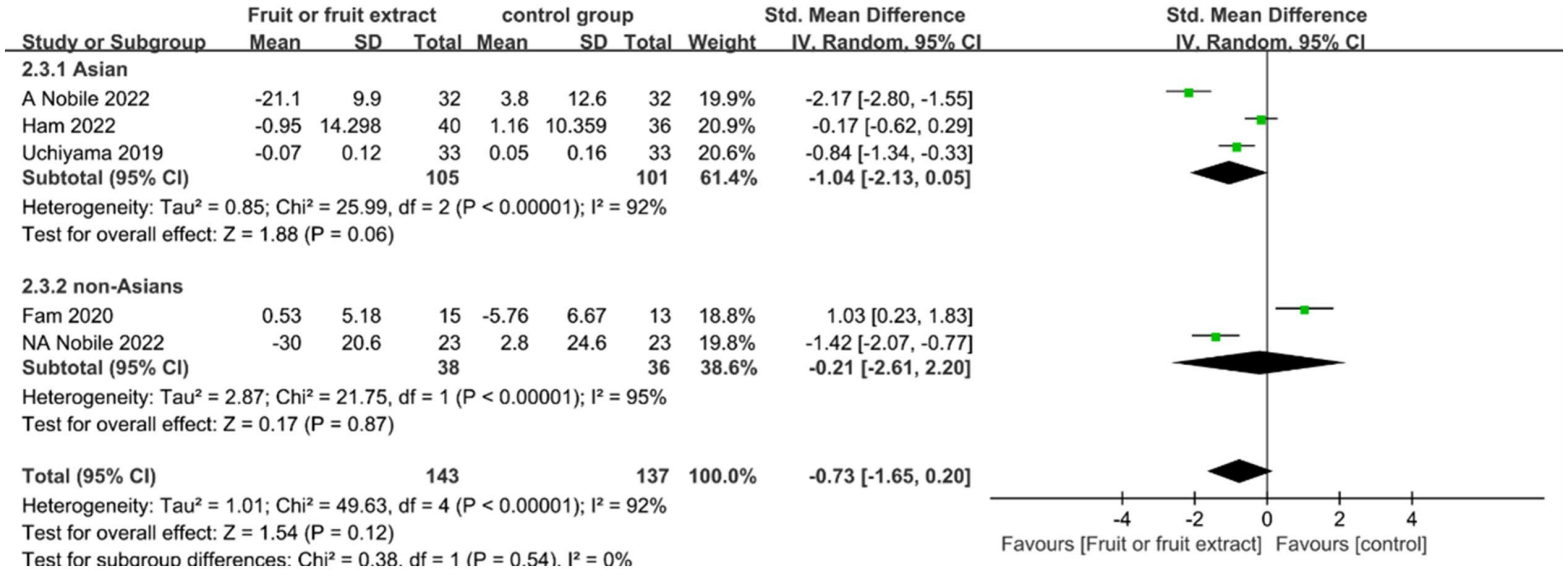
Forest plot of comparison: Fruit or fruit extract vs. placebo on wrinkle depth (SMD). The details of each study are reported in Supplementary Table S2. CI, confidence interval.

Figure 5 displays the forest plot of a Meta-analysis of four studies, which estimates the difference in wrinkle depth between intervention and placebo groups.

Among the five studies, a total of 280 participants were included, and no significant improvement in wrinkle depth was observed after consuming fruit or fruit extracts (SMD = -0.73; 95% CI: −1.65-0.20, *p* = 0.12) (Figure 5). No significant correlation was also observed for subgroup analysis: Asian group (SMD = −1.04; 95% CI: −2.13-0.05, *p* = 0.06) or non-Asian group (SMD = −0.21; 95% CI: −2.61-2.20, *p* = 0.87). However, both analyses showed significant heterogeneity between studies (I^2^ = 92 and 95%). Sensitivity analysis using the one-by-one exclusion method found that the results of the study changed when Fam 2020 was removed, and a significant improvement in skin wrinkle depth was observed after ingesting fruit extracts (SMD = -1.13; 95% CI: −1.97--0.28, *p* = 0.87) (*I*^2^ = 89%) (Supplemental Figure S3). This may be because the Fam 2020 study used a low-dose placebo group and a high-dose experimental group, while the rest of the studies used a placebo control group. Nevertheless, high heterogeneity still existed, possibly due to the complex composition of the supplement products, different extraction methods for the extracts, and differences in dosages between studies.

### Minimum erythema dose

MED was measured in two studies (*n* = 89), one with fruit extract intake (27) and the other with fruit intake (28). Both studies included participants from non-Asian groups and reported changes in MED after 8–12 weeks of fruit (27) or fruit extract (28) consumption.

**Figure 6:**
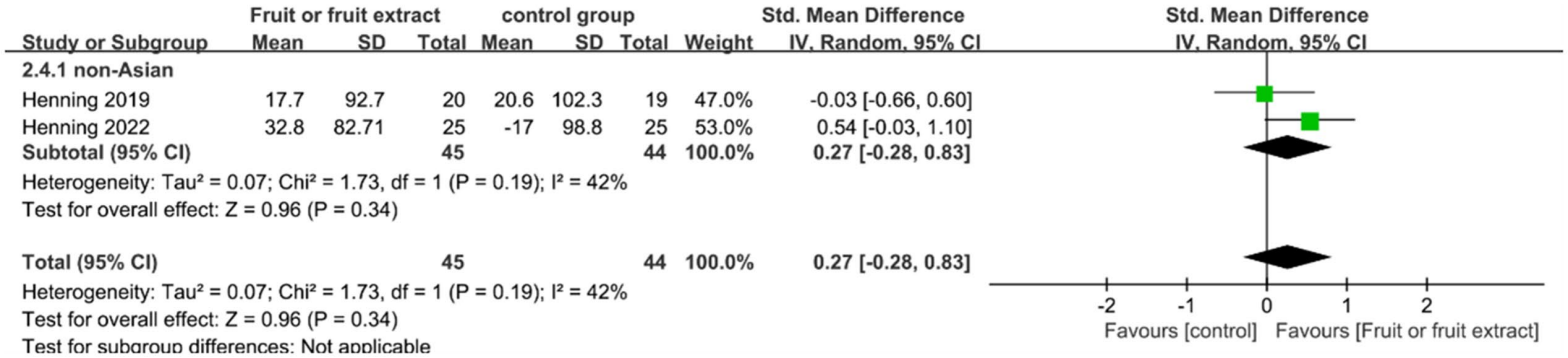
Forest plot of comparison: Fruit or fruit extract vs. placebo on MED (SMD). The details of each study are reported in Supplementary Table S2. CI, confidence interval.

Figure 6 displays the forest plot of a Meta-analysis of four studies, which estimates the difference in MED between intervention and placebo groups.

Among 89 participants in two studies, oral consumption of fruit or fruit extracts showed no statistical difference in MED compared to placebo (SMD = 0.27; 95% CI: −0.28-0.83; *p* = 0.34) (Figure 6). Meta-analysis showed moderate heterogeneity (I2 = 42%), largely due to a limited number of randomized controlled trials and the fact that the supplements consumed included both fruit extracts and whole fruits.

### Transepidermal water loss

TEWL was measured in two studies (*n* = 186), one of which was conducted simultaneously in China and Italy and was considered as two independent studies. A total of 2 studies were included in the Asian group (24, 25) and 1 study was included in the non-Asian group (24). All studies investigated the effects of fruit extracts for a duration of 8 to 12 weeks.

**Figure 7:**
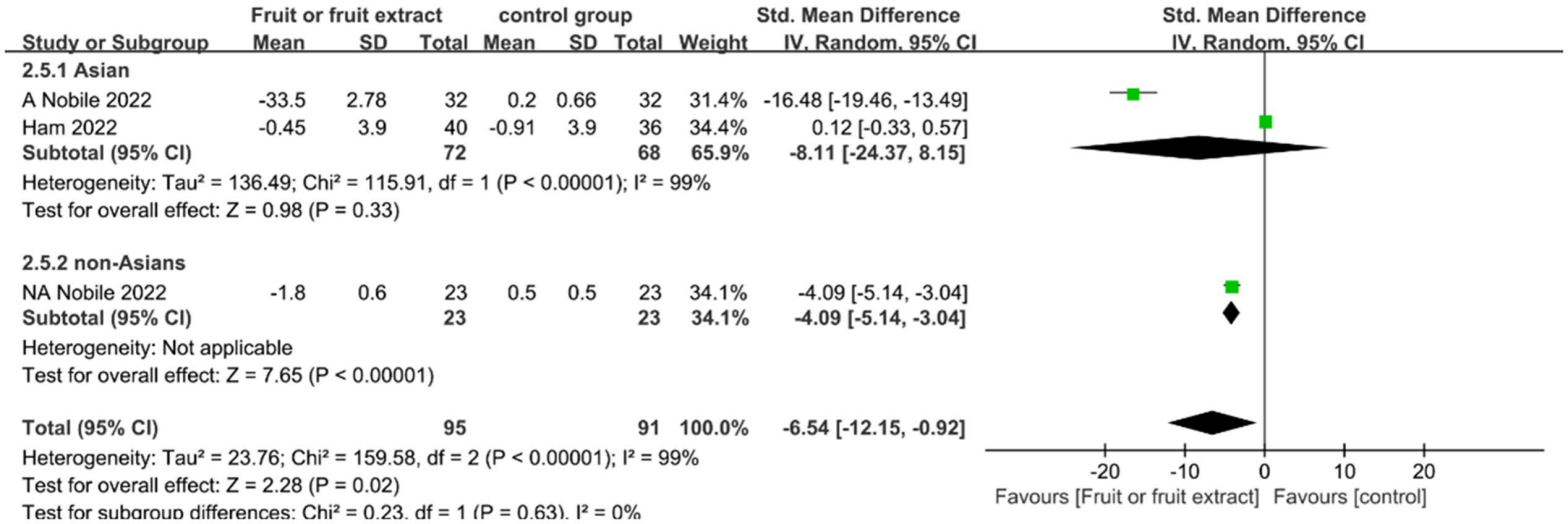
Forest plot of comparison: Fruit or fruit extract vs. placebo on TEWL (SMD). The details of each study are reported in Supplementary Table S2. CI, confidence interval.

Figure 7 displays the forest plot of a Meta-analysis of three studies, which estimates the difference in Transepidermal water loss (TEWL) between intervention and placebo groups.

A Meta-analysis of these three studies showed a significant difference between the fruit extract group and the placebo group (SMD = −6.54; 95% CI: −12.15- -0.92; *p* = 0.02) (Figure 7), indicating that the intake of fruit extracts has the ability to reduce TEWL. There was no significant difference in the change in TEWL for the Asian group (SMD = −8.11; 95% CI: −24.37-8.15; *p* = 0.33); the non-Asian group showed a significant improvement in TEWL (SMD = −4.09; 95% CI: −5.14- -3.04; *p* < 0.00001).However, there was high heterogeneity between the two studies, possibly due to differences in the tested areas (back, eye area), different types of fruit extracts (qingken orange extract, red orange (*Citrus sinensis* (L.) Osbeck) extract), and variations in extract preparation methods (standardized extract, 50% ethanol extract).

## Discussion

This study selected seven randomized controlled trials investigating the effects of oral consumption of fruits or fruit extracts on skin aging, involving a total of 387 healthy subjects. The main outcome measures were skin hydration, skin elasticity, wrinkle depth, minimum erythema dose (MED), and transcutaneous water loss (TEWL), to assess the overall impact of fruit or fruit extract intake on skin aging. Prior to this study, no Systematic review or Meta-analysis had been published specifically targeting fruits or fruit extracts. This study fills this gap by providing a comprehensive review and Meta-analysis, updating the evidence for the safety and efficacy of oral fruits and fruit extracts in improving skin aging.

Through Meta-analysis and systematic evaluation, we found that intake of fruits or fruit extracts can effectively improve skin aging in a number of ways, including skin hydration and transcutaneous water dissipation (TEWL). Skin hydration refers to the ability of the outermost layer of the skin – the stratum corneum or its degradation products – to bind to water (30). Many factors can influence the rate and extent of skin penetration, and skin hydration is one of the main factors that affect the rate and extent of skin absorption (31). Transcutaneous water loss (TEWL) is the loss of water from deep skin tissue through evaporation and diffusion across the epidermis. It is a form of skin water loss invisible to the naked eye, and its value reflects the amount of moisture evaporated from the skin surface and is an important parameter for assessing skin barrier function (32). The smaller the value of this parameter the better it is for skin barrier function and repair.Other outcome measures in the study included skin elasticity, wrinkle depth, and minimum erythema dose (MED). Ingestion of fruits or fruit extracts did not improve these skin conditions. The data on skin elasticity in this study mainly used the overall skin elasticity (R2) (33, 34), which provides a comprehensive picture of changes in skin elasticity. The observed indicator for wrinkles is wrinkle depth (35), as this indicator was clearly documented in the included literature and is more readily available.MED is the minimum UV radiation dose (Joules/cm2) or minimum exposure time required to produce the lowest erythema dose, and this indicator varies depending on individual skin photosensitivity and is referred to as phototype. This metric mainly assesses the effects of skin photoaging (36).

The mechanism of action of fruits and fruit extracts on the skin is complex and involves the presence of various nutrients and phytochemicals such as flavonoids, polyphenols, vitamins, alkaloids, anthocyanins, hydroxycinnamic acid, flavanones and ascorbic acid, which form antioxidants and other beneficial substances in the body’s cellular system (37) and exert UV protection (38) and anti-inflammatory (39) roles. Studies in humans have shown that they are effective in reducing oxidative stress in people exposed to air pollution (40) and provide photoprotection against UV-induced erythema (41) and photoaging (42–44). Several studies have shown that the nutrients in fruits and fruit extracts promote the synthesis of collagen and elastin fibers and improve skin elasticity and firmness (45, 46), which includes various vitamins, with vitamin C showing a predominance, as vitamin C has strong redox properties and is essential for maintaining the active forms of prolyl and lysyl hydroxylases. Hydroxylation of proline and lysine is carried out by prolyl hydroxylase using ascorbic acid as a cofactor. Ascorbic acid deficiency leads to reduced hydroxylation of proline and lysine, which affects collagen synthesis (47). At the same time, additional studies have demonstrated that specific phytochemicals found in fruits and fruit extracts can inhibit the activity of tyrosinase, leading to a reduction in melanin formation and consequent skin whitening effects (48). As for the bioavailability of nutrients in fruits, it may be influenced by various factors, such as the type of nutrient, fruit variety, ripeness, preparation method and interaction with other food components (49). In general, fruits are a good source of various vitamins, minerals, fiber and phytochemicals. These nutrients are usually well absorbed by the body when consumed in whole form because the fiber in fruit slows digestion and promotes absorption of the best nutrients. For example, the bioavailability of vitamin C may be better when fruit is eaten raw rather than cooked, because heating degrades this nutrient (50). On the other hand, the bioavailability of certain phytochemicals, such as lycopene in tomatoes, can be enhanced when fruits are cooked or processed because heat helps break down plant cell walls and release nutrients (51). This article deals only with the consumption of fruits and fruit extracts without cooking or special processing. Overall, as natural plant-derived substances ingested as food, they have been shown to be safe for long-term intake, and the mechanisms of action of fruits and fruit extracts on the skin need further study.

This Systematic review and Meta-analysis has several limitations. Although the search strategy was comprehensive, some clinical trials may not have been identified. Our systematic and detailed search strategy should help identify all trials and reduce bias. Another limitation is that our standardized quality assessment and data analysis was based on the information and data reported in the study rather than the study itself. Although this study confirms the role of fruit consumption and fruit extracts in improving skin aging, the number of randomized controlled trials included in this article is small and skin aging is multifactorial, and the credibility of the study may be confounded by factors such as geographic climate. Therefore, further validation and refinement of this finding will require longer randomized controlled trials, larger sample sizes, and the use of objective dermatological methods to obtain more reliable results related to the effects of fruits and fruit extracts on skin health.

Most of the included trials did not mention adverse events (AEs), thus avoiding safety concerns. As we have shown, some trials did not include the necessary information on fruit extraction and origin that would be needed to properly compare trials in terms of effectiveness. Researchers studying such products should place greater value the importance of AE reporting

## Conclusion

Overall, fruits or fruit extracts were significantly associated with skin aging in some aspects compared to placebo, but still no significant improvements were observed in many aspects. Further exploration of the magnitude of the effects of fruits and fruit extracts, especially in skin elasticity, wrinkle depth, and minimum erythema dose (MED), needs to be investigated through larger-scale and more rigorous studies. Many of the included studies had small samples and lacked satisfactory quality in terms of design and methodology. Future studies must ensure that trials are conducted and reported using methods that minimize bias as much as possible, and comply with the CONSORT statement on clinical trial reporting (52). We believe that as people’s attention to skin aging deepens and such trials continue to be conducted, more and more evidence will emerge to support the positive effects of consuming fruits and fruit extracts on skin aging-related indicators.

Currently, only two observations are valid, including skin hydration and transcutaneous water dissipation (TEWL), while the evidence for recommendations against other aspects remains insufficient.

## Data availability statement

The original contributions presented in the study are included in the article/Supplementary material, further inquiries can be directed to the corresponding author.

## Author contributions

HL, LW, JF, and LJ were suitable for the study design, literature searches, statistical analysis, and manuscript preparation. The study was supervised by JW. All authors contributed to the article and approved the submitted version.

## Funding

This work is supported by Key Research and Development Program (Major Science and Technology Projects) Fund of Sichuan Provincial Department of Science and Technology (no. 2022YFS0416) and Scientific and Technological Research Special Project of Sichuan Provincial Administration of Traditional Chinese Medicine (no. 2022CP1336).

## Conflict of interest

The authors declare that the research was conducted in the absence of any commercial or financial relationships that could be construed as a potential conflict of interest.

## Publisher’s note

All claims expressed in this article are solely those of the authors and do not necessarily represent those of their affiliated organizations, or those of the publisher, the editors and the reviewers. Any product that may be evaluated in this article, or claim that may be made by its manufacturer, is not guaranteed or endorsed by the publisher.
