# Supplementary material for: Effects of oral intake fruit or fruit extract on skin aging in healthy adults: a systematic review and Meta-analysis of randomized controlled trials
Source: Front Nutr. 2023 Aug 4;10:1232229. doi: 10.3389/fnut.2023.1232229 (PMC10436291; doi:10.3389/fnut.2023.1232229)
Supplement: Supplementary file 1 [file Data_Sheet_1.docx]

**Supplementary Material**

**Supplemental Table S1**. Search strategy used for this review.

| Database | Search strategy | Number of results |
| --- | --- | --- |
| Pubmed | (((((((((((((((((((((((((((((dietary supplements[MeSH Terms]) OR (food[MeSH Terms])) OR (diet[MeSH Terms])) OR (supplement[Title/Abstract)) OR (food[Title/Abstract])) OR (diets[Title/Abstract])) OR (oral[Title/Abstract])) OR (intake[Title/Abstract])) OR (ingestion[Title/Abstract])) OR (dietary[Title/Abstract])))) ) OR (Consumption[Title/Abstract])) OR (functional food[Title/Abstract])) OR (orally[Title/Abstract])) AND (((((((((((((((((((((skin aging[MeSH Terms]) OR (Epidermis[MeSH Terms])) OR (skin care[MeSH Terms])) OR (skin condition[Title/Abstract])) OR (skin barrier[Title/Abstract])) OR (Wrinkle[Title/Abstract])) OR (skin roughness[Title/Abstract])) OR (Aging, Skin[Title/Abstract])) OR (Solar Aging of Skin[Title/Abstract])) OR (Photoaging of Skin[Title/Abstract])) OR (Skin Wrinkling[Title/Abstract])) OR (Skin Wrinklings[Title/Abstract])) OR (Wrinkling, Skin[Title/Abstract])) OR (UVB[Title/Abstract])) OR (skin[MeSH Terms])) ) OR (anti-ageing [Title/Abstract] ))))) OR (photoaged facial skin[Title/Abstract])))) OR (skin health[Title/Abstract]))) AND ((((((((((((((((Fruit[MeSH Terms]) OR (Citrus[MeSH Terms])) OR (Fruits[Title/Abstract])) OR (Berries[Title/Abstract])) OR (Berry[Title/Abstract])) OR (Fruit Juices[Title/Abstract])) OR (Fruit Juice[Title/Abstract])) OR (Juice, Fruit[Title/Abstract])) OR (Juices, Fruit[Title/Abstract])) OR (Citrus Fruit[Title/Abstract])) OR (Avocado[Title/Abstract])) OR (Citrus hystrices[Title/Abstract])) OR (hystrices, Citrus[Title/Abstract])) OR (Extract[Title/Abstract])) OR (extracts[Title/Abstract])))) NOT (((Meta-Analysis[Publication Type]) OR (Systematic Review[Publication Type])) OR (Review[Publication Type]))) NOT (mice[MeSH Terms])) NOT (rats[MeSH Terms]) AND (2000:2023[pdat]) | 795 |
| Embase | 'aging skin':ti,ab,kw OR 'senile skin':ti,ab,kw OR 'skin aging':ti,ab,kw OR 'epidermis'/exp OR 'skin care'/exp OR 'skin condition':ti,ab,kw OR 'skin barrier'/exp OR 'wrinkle'/exp OR 'skin roughness'/exp OR 'solar aging of skin':ti,ab,kw OR 'skin wrinkle':ti,ab,kw OR wrinkles:ti,ab,kw OR wrinkling:ti,ab,kw OR 'ultraviolet b radiation'/exp OR uvb:ti,ab,kw OR skin:ti,ab,kw OR 'skin health'/expAND'dietary supplement'/exp OR 'diet supplement':ti,ab,kw OR 'dietary supplements':ti,ab,kw OR 'food supplement':ti,ab,kw OR 'food'/exp OR 'diet'/exp OR 'supplementary diet':ti,ab,kw OR 'diet additive':ti,ab,kw OR oral:ti,ab,kw OR intake:ti,ab,kw OR 'ingestion'/exp OR dietary:ti,ab,kw OR consumption:ti,ab,kw OR 'functional food'/exp OR 'functional foods':ti,ab,kwAND'fruit'/exp OR 'citrus'/exp OR fruits:ti,ab,kw OR 'berry'/exp OR berries:ti,ab,kw OR 'fruit juices':ti,ab,kw OR 'fruit juice'/exp OR 'fruits juice':ti,ab,kw OR 'citrus fruit'/exp OR 'citrus fruits':ti,ab,kw OR 'fruit, citrus':ti,ab,kw OR 'avocado'/exp OR 'extract'/expAND'randomized controlled trial':de OR 'controlled clinical trial':de OR 'randomized':ti,ab OR 'placebo':ti,ab OR 'clinical trials' OR 'randomly':ti,ab OR 'clinical study':ti,ab OR 'volunteers':ti,abAND'human'/exp OR 'homo sapiens':ti,ab,kw OR 'human being':ti,ab,kw OR 'human body':ti,ab,kw OR 'human race':ti,ab,kw OR humans:ti,ab,kw2000-2023 | 962 |
| Web of science | (((((((((((TS=(dietary supplements)) OR TS=(food)) OR TS=(diet)) OR TS=(supplement)) OR TS=(diets)) OR TS=(oral)) OR TS=(intake)) OR TS=(ingestion)) OR TS=(dietary)) OR TS=(Consumption)) OR TS=(orally)）  AND(((((((((((((((TS=(skin aging)) OR TS=(Epidermis)) OR TS=(skin care)) OR TS=(skin conditions)) OR TS=(skin barrier)) OR TS=(Wrinkle)) OR TS=(skin roughness)) OR TS=(Solar Aging of Skin)) OR TS=(Photoaging of Skin)) OR TS=(Skin Wrinkling)) OR TS=(Skin Wrinklings)) OR TS=(UVB)) OR TS=(anti-ageing)) OR TS=(photoaged facial skin[)) OR TS=(skin health)）AND(((((((((((TS=(Fruit)) OR TS=(Citrus)) OR TS=(Fruits)) OR TS=(Berries)) OR TS=(Berry)) OR TS=(Fruit Juices)) OR TS=(Fruit Juice)) OR TS=(Citrus Fruit)) OR TS=(Avocado)) OR TS=(Citrus hystrices)) OR TS=(Extract)) OR TS=(extracts)AND(((((((TS=(randomized controlled trial)) OR TS=(controlled clinical trial)) OR TS=(randomized)) OR TS=(placebo)) OR TS=(clinical trials)) OR TS=(randomly)) OR TS=(clinical study)) OR TS=(volunteers) NOT((TS=(Meta-Analysis)) OR TS=(Systematic Review)) OR TS=(Review)NOT(TS=(mice)) OR TS=(rats) | (2251) |
| Cochrane | ((MeSH descriptor: [Skin Aging] explode all trees) OR (MeSH descriptor: [Epidermis] explode all trees) OR (MeSH descriptor: [Skin Care] explode all trees) OR ("wrinkling"):ti,ab,kw OR (Skin):ti,ab,kw OR (Skin Wrinklings):ti,ab,kw OR (Skin Wrinkling):ti,ab,kw OR (Photoaging of Skin):ti,ab,kw OR (Aging):ti,ab,kw OR ("skin"):ti,ab,kw OR (Solar Aging of Skin):ti,ab,kw OR ("UVB"):ti,ab,kw OR (skin health):ti,ab,kw OR (skin condition):ti,ab,kw OR (skin barrier):ti,ab,kw OR (skin roughness):ti,ab,kw)AND ((MeSH descriptor: [dietary supplements] explode all trees) OR (MeSH descriptor: [Food] explode all trees) OR (MeSH descriptor: [Diet] explode all trees) OR (MeSH descriptor: [supplement] explode all trees)OR(MeSH descriptor: [functional food] explode all trees) OR (oral):ti,ab,kw OR (intake):ti,ab,kw OR (dietary):ti,ab,kw OR (diets):ti,ab,kw OR (ingestion):ti,ab,kw OR ("consumption"):ti,ab,kw OR (functional foods):ti,ab,kw)AND((MeSH descriptor: [fruit] explode all trees) OR (MeSH descriptor: [citrus] explode all trees) OR (MeSH descriptor: [Oils, Volatile] explode all trees) OR (MeSH descriptor: [Persea] explode all trees) OR (fruits):ti,ab,kw OR (berry):ti,ab,kw OR (fruit juice):ti,ab,kw OR ('fruit juice):ti,ab,kw OR (Avocado):ti,ab,kw OR (Avocados):ti,ab,kw OR ("extract"):ti,ab,kw)AND(MeSH descriptor: [Humans] explode all trees) OR(Homo sapiens):ti,ab,kw OR (Human):ti,ab,kw | (786) |

**Supplement Table S2.** Full details of all studies included in systematic review.

| **Study Location** | **Study Population** | | | **Intervention** | **Control** | **Formulation** | **Study Duration** | **Test**  **Conditions ( °C R.T.) (% R.H.)** | **Outcome** | | **Adverse Effects** |
| --- | --- | --- | --- | --- | --- | --- | --- | --- | --- | --- | --- |
|  | **Sample size, sex** | **Age, years** | **Health Condition** | **Contents, daily dose** | **Contents, daily dose** |  |  |  | **Meaurement Instrument** | **Parameter (Measuring Sites)** |  |
| Nobile2022(8);  China and Italy | n=110;  60% F | 35-55 | Healthy;mild to moderate skin aging signs | 1capsule :Red Orange  Complex | 1capsule :maltodextrin | capsule | 57d | NR | Corneometer CM 825, a model 601–300W solar simulator, Cutometer MPA 580 , Tewameter TM 300, Primos | back | AE=0 |
| Ham2022(8); Korea | n=76;100% F | 40-60 | Healthy,dry skin,with periorbital wrinkles | Extract of green mandarins two tablets | dextrin and crystalline cellulose two tablets | tablets | 12w | 30±22°C, 2±50% | VISIA CR, Corneometer CM 825, Corneometer CM 825, Cutometer MPA580 | periocular | NR |
| Chakkalakal2022(8); USA | n=18;83% F | 25–55 | Healthy | 75mg punicalagin | oral placebo | capsule | 4w | NR | Vapometer®,The SkinColorCatch® | forehead | AE=0 |
| Uchiyama2019(5); Japan | n=66;100% F | 35-50 | Healthy,low cheek  elasticity | 50mg of dry lingonberry extract and 60 mg of dry amla fruit extract | the sweetener, acidulant, and flavor as the test | beverages | 12W | 21±2◦C, 45%±5% | Cutometer dual MPA 580, 20-MHz ultrasound device, Corneometer CM 825, Tewameter TW210 | cheek | NR |
| Henning2019(14); USA | n=50;100% F | 30–45 | Healthy,Fitzpatrick skin type II-IV | PomX 1000 mg | placebo capsules | capsules | 12W | NR | Cutometer dual MPA580, exameter MA18, orneometer CM825 and sebumeter SM815, | arm | NR |
| Henning2022(8); USA | n=39;100% F | 27–73 | Healthy,Fitzpatrick skin type  II-IV | one avocado | continue habitual diet | fruit | 8w | NR | The cutometer | forehead | NR |
| Fam2020(5); USA | n=28;100% F | 54-66 | healthy postmenopausal women w | 250 g of Ataulfo mango, | 85g Ataulfo mango, | frozen mangos | 16w | NR | Mini Research 3D Clarity System | periocular | NR |

F = female; NR = not reported; AE = adverse events; it = intervention; pl = placebo; TAE = treatment-related adverse effect; TWD = treatment-related witdrawals;


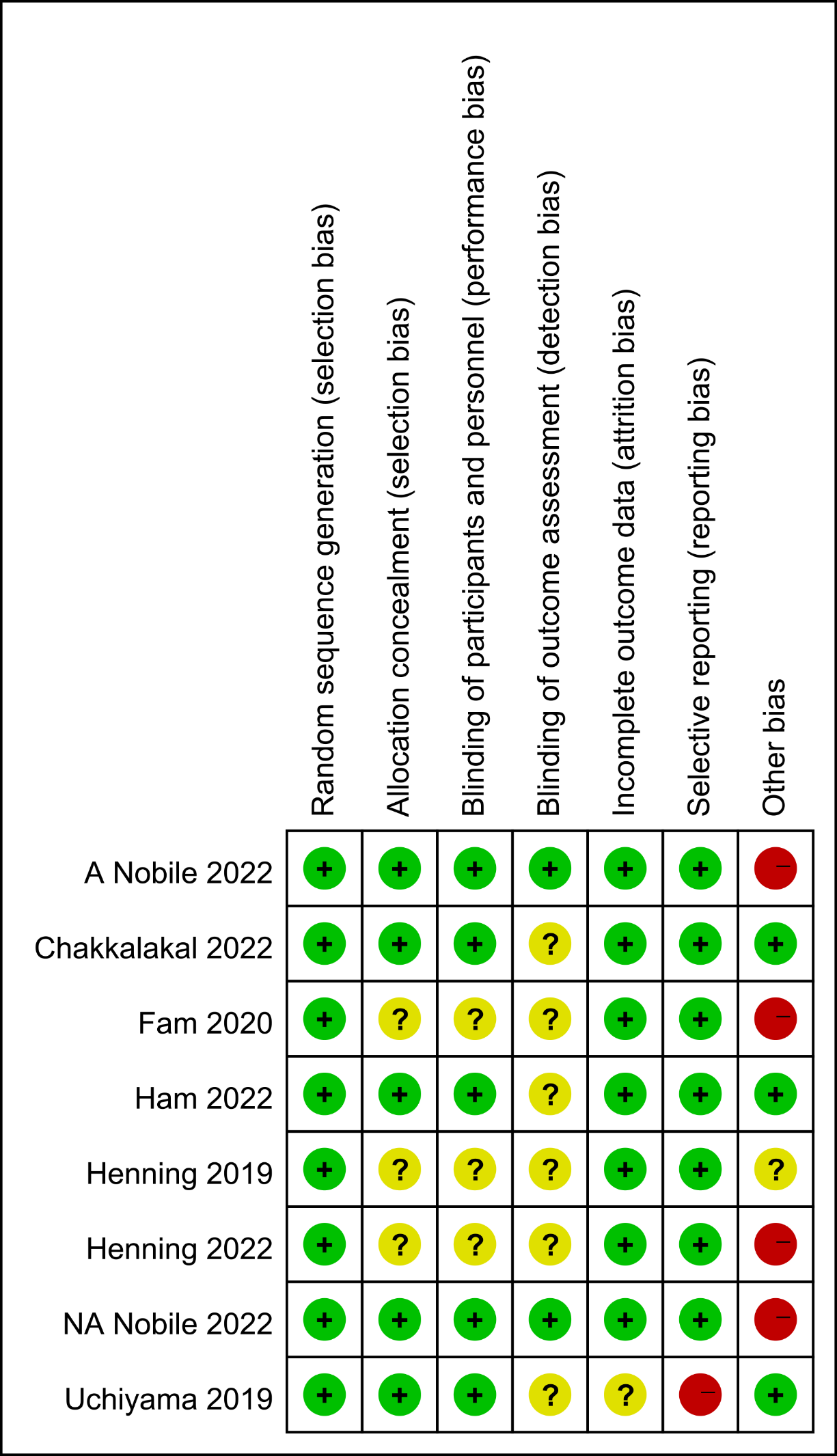


**Supplemental Figure S1** Risk of bias summary: review authors’ judgements about each risk of bias domain for each included study. + = low risk of bias; ? = unclear risk of bias; - = high risk of bias


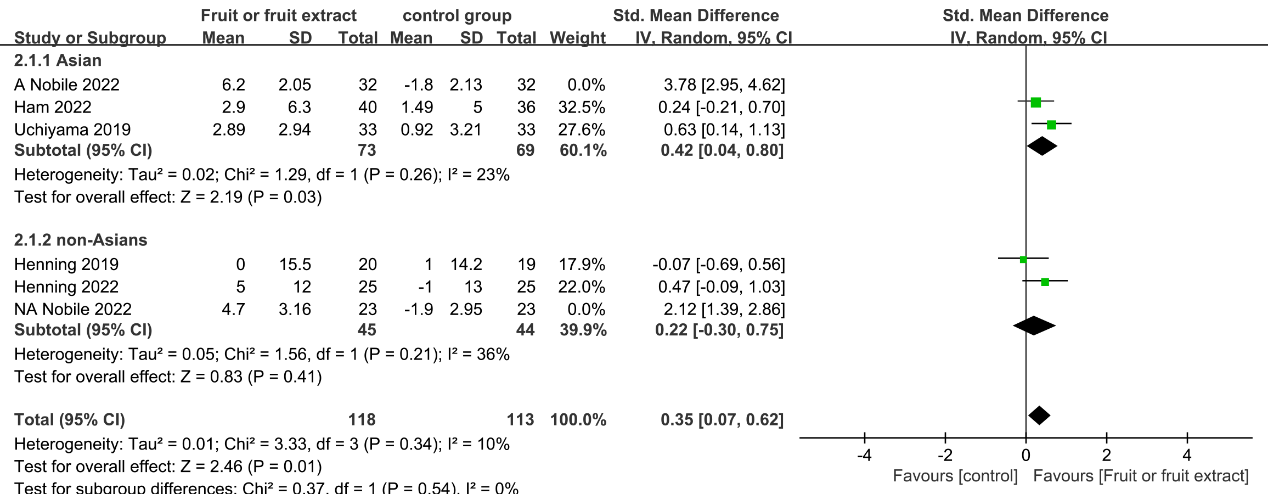


**Supplemental Figure S2**| Forest plot of comparison: Fruit or fruit extract vs. placebo on skin hydration (SMD) After excluding Nobile 2022. CI,confidence interval.


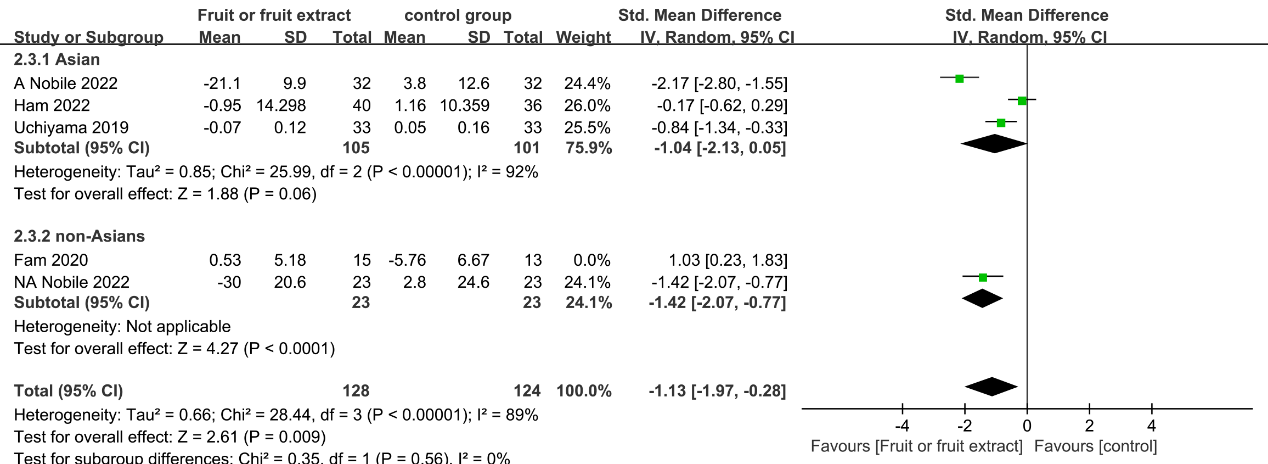


**Supplemental Figure S3**| Forest plot of comparison: Fruit or fruit extract vs. placebo on skin hydration (SMD) After excluding Fam 2020. CI,confidence interval.
